# Changes in serum β‐synuclein precede blood biomarkers of Alzheimer pathology in Down syndrome

**DOI:** 10.1002/alz.71860

**Published:** 2026-09-21

**Authors:** Alba Cervantes González, Alejandra O Morcillo‐Nieto, Sara Serrano, Bessy Benejam, Laura Videla, Isabel Barroeta, Susana Fernández, Laura del Hoyo Soriano, Aida S Hernandez, Lucía Maure‐Blesa, Lídia Vaqué‐Alcázar, Javier Arranz, Íñigo Rodríguez‐Baz, Mateus R Aranha, Danna Perlaza, Laia Lidón, Daniel Alcolea, Alexandre Bejanin, María Carmona‐Iragui, Juan Fortea, Alberto Lleó, Markus Otto, Patrick Oeckl, Olivia Belbin

**Affiliations:** ^1^ Memory Unit, IR SANT PAU Hospital de la Santa Creu i Sant Pau Barcelona Spain; ^2^ Centro de Investigación Biomédica en Red en Enfermedades Neurodegenerativas (CIBERNED) Madrid Spain; ^3^ Institut de Neurociències Universitat Autònoma de Barcelona Barcelona Spain; ^4^ Barcelona Down Medical Center Fundació Catalana Síndrome de Down Barcelona Spain; ^5^ Department of Medicine, Faculty of Medicine and Health Sciences Institute of Neurosciences University of Barcelona Barcelona Spain; ^6^ Institut d'Investigacions Biomèdiques August Pi i Sunyer (IDIBAPS) Barcelona Spain; ^7^ Department of Neurology Martin‐Luther‐University Halle‐Wittenberg Halle (Saale) Germany; ^8^ Department of Neurology University Hospital Ulm Ulm Germany; ^9^ German Center for Neurodegenerative Diseases (DZNE) Ulm Ulm Germany

**Keywords:** Alzheimer's disease, beta‐synuclein, blood biomarkers, Down syndrome

## Abstract

**INTRODUCTION:**

There is a need for early, objective markers of Alzheimer's disease (AD)‐related synapse dysfunction in adults with Down syndrome (DS). The presynaptic protein β‐synuclein is elevated in the blood of adults with DS. This study evaluates the positioning of these changes relative to changes in pathophysiological blood biomarkers along the AD continuum.

**METHODS:**

We quantified serum β‐synuclein using immunoprecipitation–mass spectrometry in a cross‐sectional cohort (*n* = 131) spanning the AD continuum in adults with DS (*n* = 88) and cognitively unimpaired euploid controls (*n* = 43).

**RESULTS:**

β‐synuclein levels were elevated in individuals with DS (*p* < 0.001), preceding symptom onset by two decades and changes in other blood biomarkers (tau phosphorylated at threonine 217, neurofilament light, glial fibrillary acidic protein) by several years. Higher β‐synuclein was associated with cortical atrophy (*p* < 0.001) and hypometabolism (*p* < 0.001) in AD‐vulnerable regions and reduced episodic memory (*p* < 0.009).

**DISCUSSION:**

These findings consolidate β‐synuclein as an early blood‐based biomarker of synaptic dysfunction and provide insight into early AD‐related mechanisms.

## Highlights

- Serum β‐synuclein rises decades before AD symptoms in DS.
- β‐synuclein changes precede pTau217, NfL, and GFAP alterations.
- Higher β‐synuclein associates with cortical atrophy and hypometabolism.
- β‐synuclein levels correlate with reduced episodic memory performance.
- β‐synuclein is an early blood marker of AD‐related synaptic dysfunction.

## 1 BACKGROUND

Down syndrome (DS) is a priority population to study blood‐based biomarkers for Alzheimer's disease (AD). Emerging blood biomarkers of AD pathophysiology (tau protein phosphorylated at threonine 181 [pTau181] and threonine 217 [pTau217]),^1^, ^2^ axonal degeneration (neurofilament light chain [NfL]), astrogliosis (glial fibrillary acidic protein [GFAP]) and synapse degeneration (β‐synuclein) reflect AD‐associated alterations in DS and have shown good diagnostic performance to diagnose symptomatic AD in people with DS.^3^, ^4^, ^5^, ^6^ The median age of prodromal AD diagnosis (i.e., age at expected AD onset) in DS is 50 years, with a median onset of dementia at 54 years.^4^ Symptomatic AD prevalence increases with age, reaching 90%–100% in the seventh decade of life.^4^

β‐synuclein is a presynaptic protein that is highly expressed in the temporal lobe^7^ and enriched in VGLUT1‐positive glutamatergic synapses.^8^ While not specific to AD,^9^ β‐synuclein is reduced in brains with AD pathology^8^ and elevated in both CSF and blood of AD patients.^9^, ^10^, ^11^ β‐synuclein is a promising biomarker of early AD changes in adults with DS.

When comparing across studies, a picture emerges whereby serum levels of β‐synuclein increase in adults with DS at approximately 27 years.^6^ This is followed by plasma NfL in the fourth decade of life, later increases in plasma and cerebrospinal fluid (CSF) pTau181 in the mid‐to‐late fourth decade and amyloid positron emission tomography (PET) uptake in the late fourth and the fifth decade^1^, ^3^, ^4^. ^18^F‐fluorodeoxyglucose (FDG) PET uptake decreases linearly from the beginning of the fourth decade, whereas hippocampal atrophy occurs later in the fifth decade, closely followed by changes in cognition in the fifth decade of life^1^, ^3^, ^4^. Thus, serum β‐synuclein is one of the earliest biomarkers, together with CSF Aβ and plasma NfL, years before changes in plasma or CSF pTau or amyloid PET uptake in DS. However, a direct comparison of serum β‐synuclein with other biomarkers within the same study across the whole AD continuum in DS is lacking.

The primary objective of this study was to assess the utility of β‐synuclein as an early indicator of AD‐related cognitive decline in adults with DS and to examine its relationship with established biofluid and neuroimaging biomarkers. We h assessed serum β‐synuclein in the Down Alzheimer Barcelona Neuroimaging Initiative (DABNI) cohort of adults with DS,^12^ which includes participants across the AD continuum (preclinical, prodromal, and AD dementia stages) and cognitively unimpaired controls. We compared β‐synuclein with established blood biomarkers (pTau217, NfL, GFAP) reflecting AD pathology, axonal degeneration, astrogliosis, and synaptic degeneration and showed a correlation between β‐synuclein and neuroimaging biomarkers (cortical volume and glucose metabolism), as well as with cognitive performance, across preclinical, prodromal, and dementia stages of AD in DS. Our findings consolidate β‐synuclein as a promising early surrogate blood‐based biomarker of AD‐related changes in this population.

## 2 METHODS

### 2.1 Study design

This is a single‐center, cross‐sectional study of serum levels of β‐synuclein in adults with DS and cognitively unimpaired controls. The study was approved by the Institutional Review Board at IR SANT PAU and was conducted in accordance with the Declaration of Helsinki. Euploid controls were selected from the Sant Pau Initiative in Neurodegeneration (SPIN) cohort, a prospective longitudinal cohort at Hospital Sant Pau, Barcelona, Spain.^13^ Adults with DS were selected from DABNI, a prospective longitudinal cohort, linked to a population‐based health plan in Catalonia, Spain, led by the Fundació Catalana Síndrome de Down and Hospital de Sant Pau.^3^, ^4^, ^12^ Inclusion criteria for controls required the absence of a cognitive or neurological disorder and normal CSF core AD biomarker (amyloid beta [Aβ]_42/40_ ratio, total tau, pTau181) concentrations using our validated cut‐offs for sporadic AD.^14^ For adults with DS, inclusion criteria for participation in the study required that all participants be over 18 years of age. Where consent was given, participants received a comprehensive neurological and neuropsychological evaluation and underwent a lumbar puncture to assess CSF biomarkers. As in previous studies, participants with DS were classified by neurologists and neuropsychologists, blind to biomarker data, in a consensus meeting into asymptomatic AD (aDS), prodromal AD (pDS), and AD dementia (dDS) according to previously published criteria.^3^

### 2.2 Neuropsychological assessment

**RESEARCH IN CONTEXT**

1. **Systematic review**: The authors reviewed the literature using PubMed to identify studies investigating blood‐based biomarkers of AD in adults with DS. Previous studies showed that β‐synuclein was elevated in blood and CSF in sporadic AD and in adults with DS. However, direct comparisons between β‐synuclein and established blood biomarkers across the full AD continuum within the same DS cohort are limited.
2. **Interpretation**: Our findings demonstrate that serum β‐synuclein increases early in adults with DS, preceding symptom onset and changes in established blood biomarkers by several years. Associations with cortical atrophy, hypometabolism, and reduced episodic memory further support β‐synuclein as an early marker of AD‐related synaptic dysfunction.
3. **Future directions**: Longitudinal studies are needed to determine the prognostic utility of β‐synuclein for predicting cognitive decline and disease progression in DS. Future work should also evaluate the specificity of β‐synuclein relative to other neurodegenerative conditions and assess its utility as a pharmacodynamic biomarker in clinical trials targeting early AD‐related synaptic dysfunction.

The level of intellectual disability (ID) was categorized according to the Diagnostic and Statistical Manual of Mental Disorders, Fifth Edition, as mild, moderate, severe, or profound ID, based on carers’ reports of the individuals’ best‐ever level of functioning.^15^ Neurological and neuropsychological examination of individuals with DS included the Cambridge Examination for Mental Disorders of Older People with DS and others with intellectual disabilities (CAMDEX‐DS), a semi‐structured clinical and informant‐based questionnaire to assess the presence of dementia symptoms, and the Cambridge Cognition Examination for Older Adults with Down syndrome (CAMCOG‐DS) adapted to evaluate multiple cognitive domains in individuals with DS, including orientation, language, memory, attention, praxis, abstract thinking, and perception. It serves as a key tool for identifying cognitive decline in this population.^15^, ^16^, ^17^ Declarative verbal memory was evaluated by the modified Cued Recall Test immediate recall phase (mCRTi) and delayed recall phase (mCRTd), as previously described.^18^

### 2.3 Biofluid collection, biomarker assessment

Serum and plasma samples were collected as previously described^19^ and stored at −80°C. Samples had not been thawed before analysis. Commercially available fully automated immunoassays were used to determine levels of plasma biomarkers: GFAP and NfL using Single Molecule Array (Quanterix) and pTau217 using Lumipulse (Fujirebio). β‐synuclein levels were measured in serum with immunoprecipitation–mass spectrometry (IP‐MS), as recently described.^20^ Because the IP‐MS assay was originally developed and validated in serum,^9^ we used the same sample matrix for β‐synuclein to maintain methodological consistency.

### 2.4 Brain imaging

A subset of participants with DS underwent a 3 Tesla (3T) magnetic resonance imaging (MRI) (*n* = 60; aDS = 26; pDS = 13; dDS = 21) and/or ^18^FDG‐PET (*n* = 42; aDS = 16; pDS = 8; dDS = 18). All participants who underwent ^18^FDG‐PET also underwent MRI. T1‐weighted images were acquired on a 3T Philips‐Achieva scanner at Hospital del Mar (Barcelona, Spain) or a 3T Siemens Prisma scanner at Hospital Clinic (Barcelona, Spain). ^18^FDG‐PET scans were acquired on an integrated PET‐computed tomography (CT) system at Hospital de la Santa Creu i Sant Pau (Barcelona, Spain) on a Philips Gemini TF or Vereos scanner. Both ^18^FDG‐PET and MRI scans were conducted within a maximum interval of 1 year. The Computational Anatomy Toolbox^21^ (CAT12) for the Statistical Parametric Mapping 12 (SPM12) toolbox was used to preprocess the structural T1‐weighted images and extract total intracranial volumes (TIV). Modulated and normalized gray matter maps were smoothed using an 8‐mm full‐width at half‐maximum (FWHM) Gaussian kernel. ^18^FDG‐PET images were spatially normalized to an ^18^FDG‐PET template, intensity‐scaled by the gray matter cerebellum, and smoothed using an 8‐mm FWHM Gaussian kernel.

### 2.5 Statistical analysis

All statistical analyses were performed in R. Group comparisons were performed using *χ* ^2^ test for categorical variables and linear models for continuous variables. Pairwise comparisons were performed using estimated marginal means with Benjamini–Hochberg correction for multiple testing. Continuous plasma biomarker measures (pTau217, NfL, GFAP, and β‐synuclein) were standardized using *z*‐score transformation (mean = 0, SD = 1) to place all biomarkers on a common scale. To study the correlation between biomarkers and age, locally estimated scatterplot smoothing (LOESS) was used to account for the non‐linear trajectory of these measurements. We defined biomarker change as the age at which the curves diverge visually (i.e., the lower age at which the 95% confidence intervals of groups did not overlap). The Spearman method was used to determine the correlation between β‐synuclein and other continuous variables. Associations between β‐synuclein and cognitive scores were assessed using linear regression models adjusted for ID severity and were restricted to individuals with mild or moderate ID. Statistical significance for all tests was set at 5% (*p* < 0.05). For the neuroimaging analysis, we used voxel‐wise multiple regressions to assess the association between β‐synuclein and both gray matter volume and glucose metabolism in the whole DS cohort, but also in aDS and symptomatic DS (sDS), comprising pDS and dDS. Analyses were performed in a mask that excluded non‐gray matter voxels, and the statistical models were corrected for TIV for models including gray matter volume. Voxel‐wise results are presented at an uncorrected threshold *p* < 0.001 together with a cluster size *k* > 100 mm^3^. This uncorrected threshold was applied given the small sample size of the cohort, particularly for the analyses stratified by clinical status, which involved smaller subgroups and were not powered for formal inference. Voxel‐wise findings are therefore considered exploratory.

## 3 RESULTS

### 3.1 Demographics

Table 1 shows the demographic and clinical data for the participants included in the study. We included 88 participants with DS and 43 cognitively unimpaired controls. The percentage of females was higher in controls compared to DS (*χ* ^2^ = 10.62, *p* = 0.001). Compared to controls, the mean age was lower in aDS (*Z* = −3.88, *p* = 0.006), comparable in pDS (*Z* = −0.73, *p* = 1.0) and in dDS (*Z* = −1.85, *p* = 0.3). There were differences in the level of ID across DS groups (*p* = 0.4). Apolipoprotein E (*APOE*) ε4 status was similar across groups (*χ* ^2^ = 18.3, *p* = 0.11).

**TABLE 1:** Demographics, clinical, and biomarker data for study participants.

Variable	Control *N* = 43^a^	aDS *N* = 35^a^	pDS *N* = 18^a^	dDS *N* = 35^a^	*p* ^b^
**Age**	46.3 (12.0, 23.0 to 65.0)	38.6 (9.1, 21.0 to 56.0)	50.4 (4.1, 46.0 to 60.0)	51.9 (5.1, 42.0 to 62.0)	<0.001
**Percentage female**	81	49	56	49	0.007
**APOE ε4 carriers**	12 (28%)	3 (8.6%)	4 (22%)	5 (13.9%)	0.10
**Percentage mild/moderate ID**	NA	88	83	74%	0.4
**CAMCOG‐DS**	NA	71.7 (15.8, 39.0 to 95.0)	64.9 (16.0, 28.0 to 85.0)	44.1 (17.2, 15.0 to 80.0)	<0.001
**mCRTi**	NA	17.9 (5.5, 0.0 to 27.0)	10.8 (3.6, 6.0 to 17.0)	4.6 (3.8, 0.0 to 14.0)	<0.001
**mCRTd**	NA	11.4 (1.0, 8.0 to 12.0)	7.6 (3.6, 0.0 to 12.0)	2.5 (2.6, 0.0 to 9.0)	<0.001
**Plasma pTau217 (pg/mL)**	0.1 (0.2, 0.1 to 1.0)	0.2 (0.2, 0.1 to 1.1)	1.2 (0.7, 0.2 to 2.7)	1.9 (1.1, 0.2 to 4.8)	<0.001
**Plasma GFAP (pg/mL)**	100.7 (74.9, 22.8 to 332.6)	108.2 (43.3, 45.5 to 196.9)	229.8 (66.9, 94.6 to 347.4)	393.2 (243.2, 134.9 to 1,365.1)	<0.001
**Plasma NfL (pg/mL)**	6.4 (4.2, 1.4 to 27.2)	10.6 (7.8, 3.4 to 37.0)	20.2 (7.7, 10.0 to 32.2)	32.4 (21.6, 10.3 to 128.7)	<0.001
**Serum β‐synuclein**	5.5 (2.2, 2.8 to 13.8)	10.1 (4.3, 4.0 to 23.3)	15.6 (6.8, 8.5 to 36.7)	20.3 (8.7, 9.6 to 42.3)	<0.001

^a^ Mean (SD, min‐max); *n* (%).

^b^ Kruskal–Wallis rank‐sum test, Pearson's chi‐squared test, Fisher's exact test.

### 3.2 Cross‐sectional age associations suggest serum β‐synuclein diverges earlier than other blood‐based biomarkers in adults with DS

**FIGURE 1:**
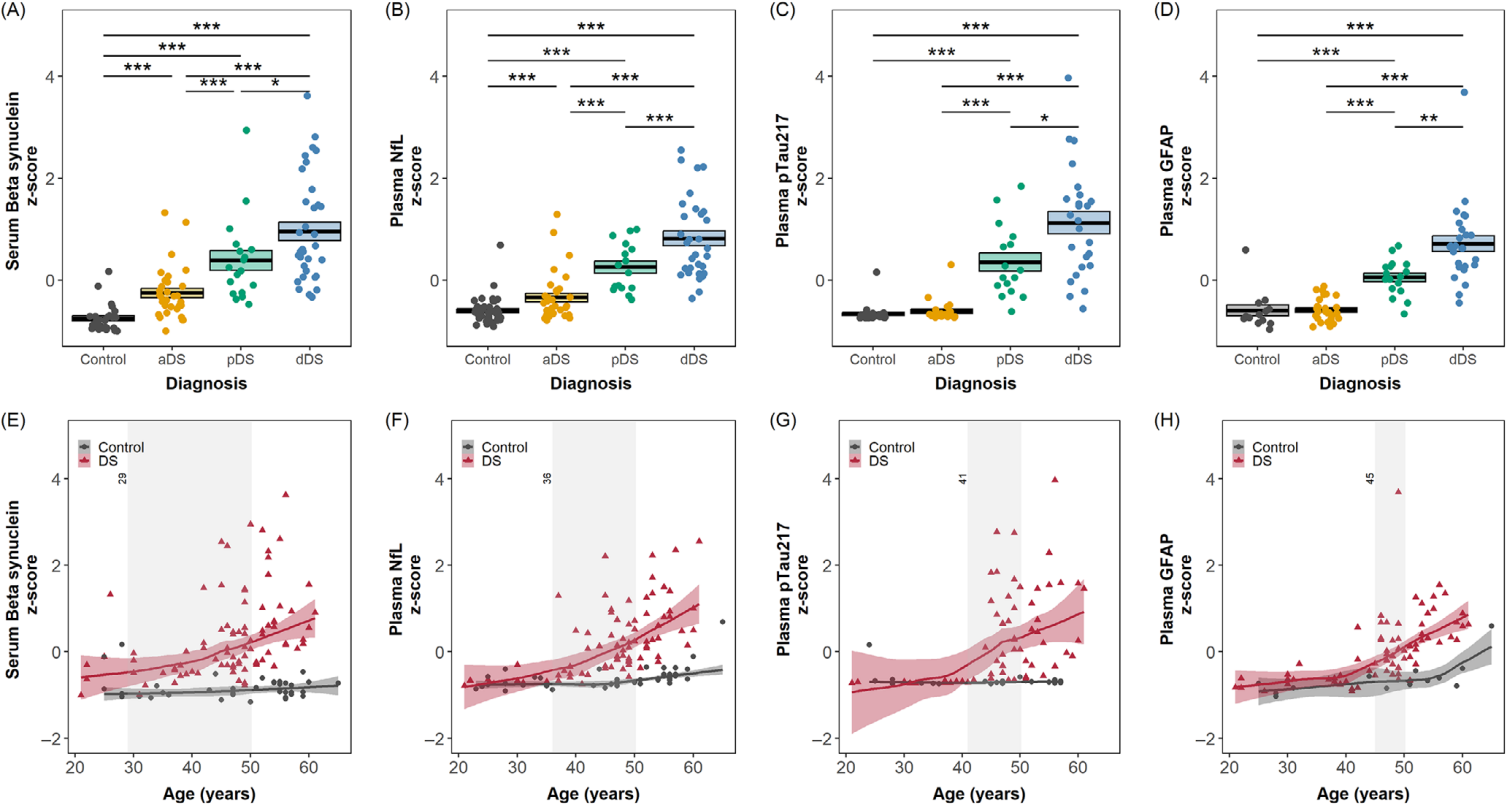
Levels of serum β‐synuclein and blood‐based biomarkers in euploid controls and adults with DS across the AD continuum. (A–F) Box plots show *z*‐score distribution (mean ± standard error of the mean) of the biomarkers quantified in blood for non‐trisomic cognitively normal subjects (controls) and adults with DS across AD stages: asymptomatic AD (aDS), prodromal AD (pDS), or AD dementia (dDS); *p < 0.05, **p < 0.01, ***p < 0.001 (pairwise comparisons from linear models with BH correction). (G–L) Age at analysis (years) is plotted against each biomarker *z*‐score in controls and adults with DS, symptomatic and asymptomatic for AD. LOESS regression lines are shown for each group (see legends). Shaded areas represent the standard error of the regression lines. The vertical gray shaded area represents the interval between the ages at which the regression lines diverge and the expected age of symptom onset (e.g., the median age of prodromal AD diagnosis).

**FIGURE 2:**
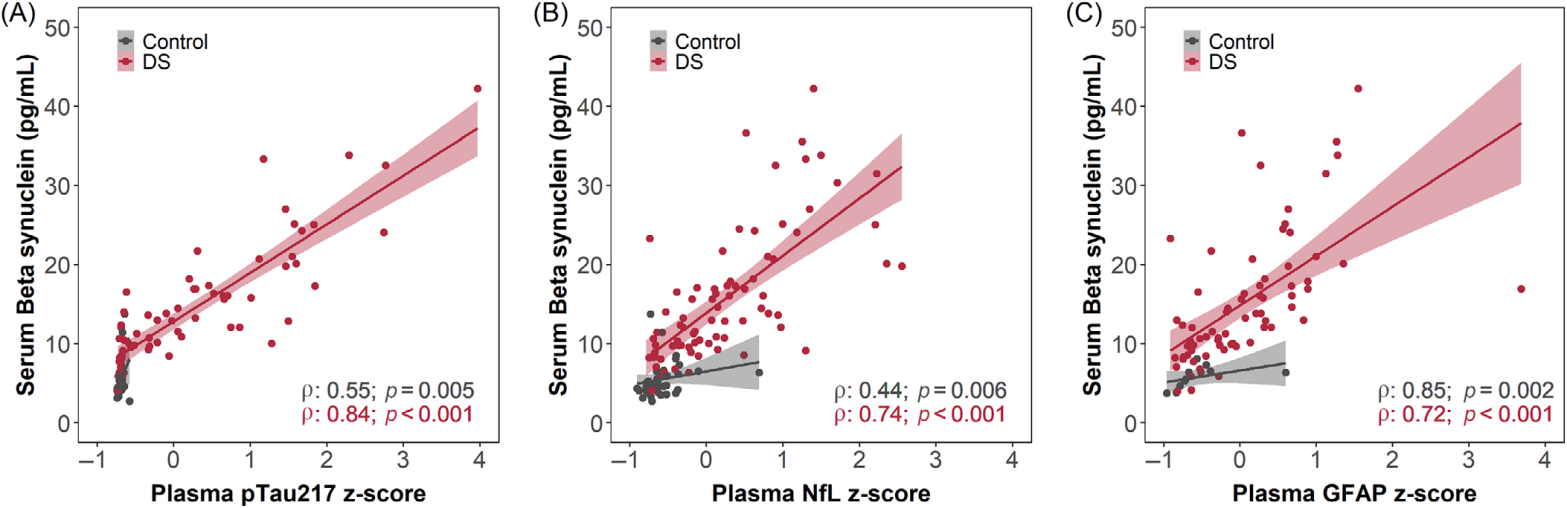
Relationship between serum β‐synuclein and blood‐based biomarkers in controls and adults with DS. Linear regression lines, coefficients (ρ), and *p* values are shown for each association, and shaded regions represent the standard error of the regression lines.

Figure 1A–D shows the median levels of serum β‐synuclein and other blood‐based biomarkers (pTau217, NfL, GFAP) in controls and adults with DS across the AD continuum. Compared to controls, serum β‐synuclein was higher in the aDS (1.83‐fold, *p* < 0.001) group and sequentially higher with advancing AD stages (pDS; 2.82‐fold, *p* < 0.001, dDS; 3.67‐fold, *p* < 0.001 vs controls). We observed a similar pattern for plasma NfL with higher levels in aDS (1.65‐fold, *p* < 0.001), pDS (3.15‐fold, *p* < 0.001), and dDS (5.04‐fold, *p* < 0.001) compared to controls. Plasma pTau217 and GFAP were higher in pDS (pTau217; 8.86‐fold, *p* < 0.001, GFAP; 2.28‐fold, *p* < 0.001) and dDS (pTau217; 14.79‐fold, *p* < 0.001, GFAP; 3.90‐fold, *p* < 0.001) compared to controls. In a subanalysis, serum β‐synuclein and plasma NfL were the only biomarkers that were already elevated (1.75‐fold, *p* < 0.001; 1.46‐fold *p* = 0.01, respectively) in aDS individuals who were negative for CSF Aβ_42/40_ ratio (aDS Aβ−) compared with controls (Figure S1). Figure 1E–H shows the association of the blood‐based biomarkers with age in DS and controls. Serum β‐synuclein was elevated in adults with DS compared to controls, with confidence intervals diverging from the third decade of life, 21 years before the average prodromal AD diagnosis in this cohort.^4^ β‐synuclein continued to increase with age with a mild inflection point of pronounced elevation that was apparent at age 40, 10 years before average symptom onset. In comparison, while confidence intervals for plasma pTau217, NfL, and GFAP in DS did not diverge from controls until the late fourth to early fifth decade, an inflection point of pronounced elevation at age 40 was apparent for all biomarkers. Figure 2 shows that β‐synuclein correlated most strongly with pTau217 (rho = 0.84), followed by NfL (ρ = 0.74) and GFAP (ρ = 0.72) in DS and with GFAP (ρ = 0.85), followed by pTau217 (ρ = 0.55) and NfL (ρ = 0.44) in controls. Figure S2 shows that serum β‐synuclein was comparable across male and female participants in the control (*p* = 0.30) and DS (*p* = 0.22) groups and between *APOE* ε4 carriers and non‐carriers in controls (*p* = 0.57) and DS (*p* = 0.66).

### 3.3 Serum β‐synuclein is associated with cognitive performance in adults with DS

**FIGURE 3:**
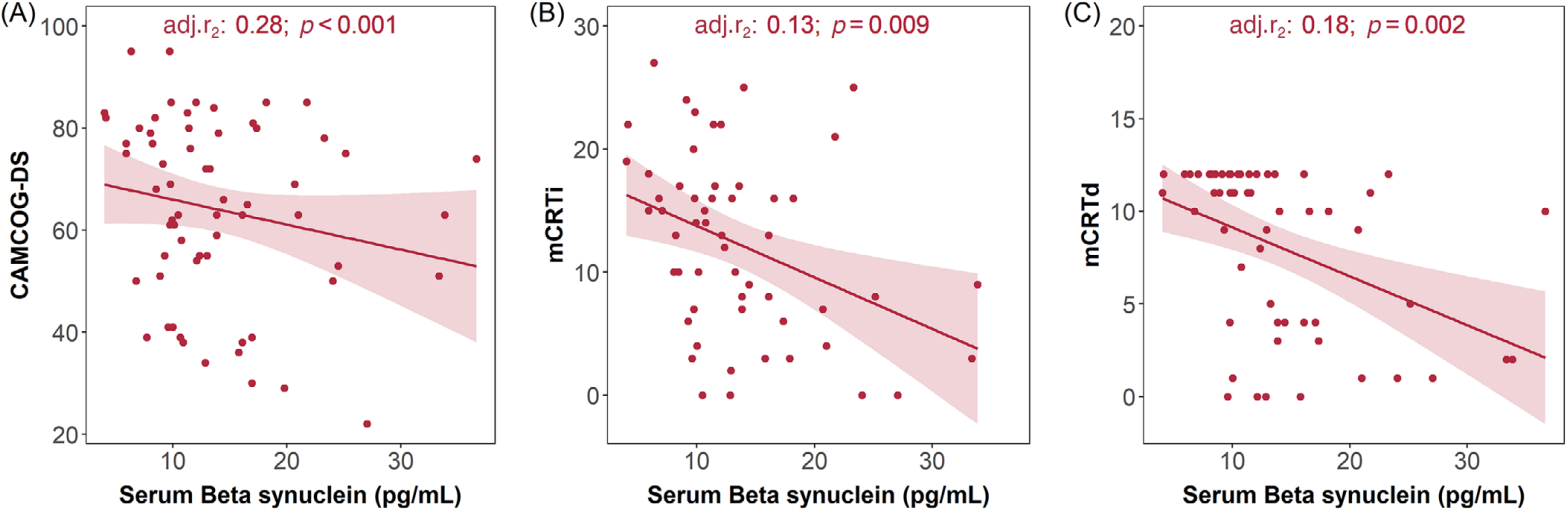
Relationship between serum β‐synuclein and measures of cognitive performance in DS. Serum β‐synuclein levels in adults with DS with mild/moderate intellectual disability are plotted against (A) CAMCOG‐DS, (B) modified cued immediate recall test (mCRTi), and (C) modified cued delayed recall test (mCRTd). Linear regression lines are shown. Shaded areas represent standard error of regression lines.

We next explored the relationship between serum β‐synuclein and the presence of dementia symptoms using the Cambridge Cognition Examination for Older Adults with Down syndrome (CAMCOG‐DS) and measures of declarative verbal memory, evaluated by the modified Cued Recall Test (mCRT) immediate recall phase (mCRTi) score and mCRT delayed recall phase (mCRTd) score, in adults with DS (see Methods). We performed regression analysis of serum β‐synuclein and cognitive performance, including the level of ID as a covariate to account for its influence on baseline cognitive performance (Figure 3). Analyses were restricted to individuals with mild to moderate ID, as the CAMCOG‑DS and mCRT are most interpretable and reliable in this population. In adults with DS, serum β‐synuclein showed modest but significant associations with CAMCOG‐DS (adj. *r*2 = 0.28, *p* < 0.001), mCRTi (adj. *r*2 = 0.13, *p* = 0.009), and mCRTd (adj. *r*2 = 0.18, *p* = 0.002). Figure S3 shows that when associations were tested separately within the aDS, pDS, and dDS groups, significant associations between serum β‐synuclein levels and CAMCOG‐DS scores were observed in aDS (adj. *r* ^2^ = 0.34, *p* = 0.002) and pDS (adj. *r* ^2^ = 0.52, *p* = 0.007) but not dDS (adj. *r* ^2^ = −0.08, *p* = 0.8). When the DS group was further divided according to the severity of ID (mild or moderate ID), associations with CAMCOG‐DS and mCRTi were maintained only in participants with mild ID: CAMCOG‐DS (adj. *r* ^2^ = 0.18, *p* = 0.03), mCRTi (adj. *r* ^2^ = 0.28, *p* = 0.01), but not in those with moderate ID (CAMCOG‐DS: adj. *r* ^2^ = −0.01, *p* = 0.40; mCRTi: adj. *r* ^2^ = 0.04, *p* = 0.12). Serum β‐synuclein was associated with mCRTd in the DS group with mild (adj. *r* ^2^ = 0.23, *p* = 0.01) and moderate ID (adj. *r* ^2^ = 0.14, *p* = 0.01).

### 3.4 Elevated serum β‐synuclein is associated with reduced cortical volume and glucose metabolism in core AD regions

**FIGURE 4:**
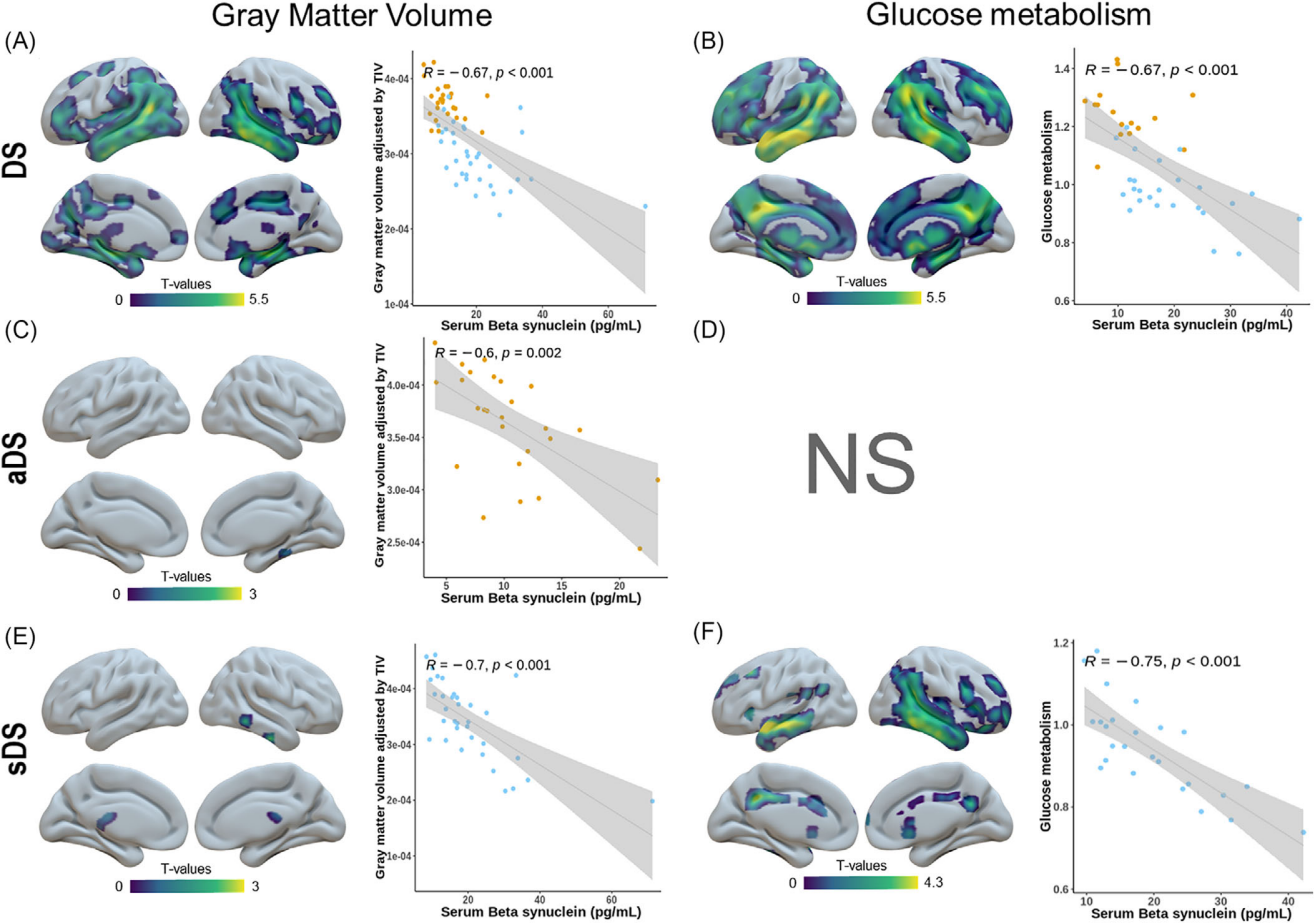
Relationship between serum β‐synuclein and neurodegeneration in Down syndrome. Brain representations show the results of the voxel‐wise regression models between either gray matter volume (left) and glucose metabolism (right), negatively associated with serum β‐synuclein in (A and B) the whole DS cohort and (C and D) aDS and (E and F) sDS. The significance of the result (*T*‐values) is showing an uncorrected threshold (*p* < 0.001, *k* > 100 mm^3^). Adjacent scatterplots display the correlation between β‐synuclein within the corresponding regional gray matter volume (left) and glucose metabolism (right) where voxel‐wise *p* < 0.05. The points represent individual patients, and the color indicates their clinical diagnosis (aDS in orange and sDS in blue). Shaded areas represent 95% confidence interval. DS; Down syndrome; aDS, asymptomatic DS; sDS, symptomatic DS (sDS).

We investigated voxel‐wise associations between serum β‐synuclein and both gray matter volume and glucose metabolism in the DS population with available neuroimaging. The pDS and dDS groups were pooled into one symptomatic group (sDS) due to reduced numbers when analyzed separately (see Methods). Increased serum β‐synuclein levels were associated with lower gray matter volume and metabolism in brain regions typically involved in AD, including the medial and lateral temporo‐parietal regions, as well as the prefrontal lobe (Figure 4). When stratifying the DS cohort by clinical status, associations with gray matter were limited to the fusiform gyrus in aDS and to the thalamus and left lateral temporal regions in sDS. For ^18^FDG‐PET, no significant associations were found in aDS. In sDS, serum β‐synuclein levels were related to glucose metabolism in the posterior cingulate and the medial and lateral temporal structures.

## 4 DISCUSSION

The main finding of this study was that serum β‐synuclein is elevated in adults with DS compared to cognitively unimpaired controls by the third decade of life and gradually increases throughout the fourth, fifth, and sixth decades. Cross‐sectional age associations suggested that changes in serum β‐synuclein were detectable before changes in CSF amyloid markers, with an estimated divergence approximately 21 years before prodromal AD and more than 7 years before other blood‐based biomarkers (pTau217, NfL, and GFAP). β‐synuclein was associated with mCRT, a test of episodic memory typically related to medial temporal lobe damage,^22^ and was associated with reduced cortical volume and glucose metabolism in regions involved in AD pathology. Taken together, we propose that early changes in serum β‐synuclein reflect underlying AD pathophysiology in adults with DS, consistent with studies that show that synaptic alterations are one of the earliest AD‐related events in DS.^23^ The early increase in β‐synuclein observed in asymptomatic, Aβ− adults with DS may reflect trisomy 21‐associated neurobiology, early AD‐related synaptic dysfunction, or both. While our study cannot distinguish between these possibilities, the associations of β‐synuclein with established AD biomarkers, cognition, cortical volume, and glucose metabolism support the hypothesis that AD‐related synaptic dysfunction contributes to the observed early changes.

Previous studies in CSF and blood had already highlighted the potential of β‐synuclein as an early synaptic marker of AD,^9^, ^11^, ^20^, ^23^, ^24^, ^25^ showing already an increase in the preclinical stages of the disease.^20^, ^26^, ^27^ In CSF, β‐synuclein is unaltered in other neurodegenerative dementias, including frontotemporal dementia, amyotrophic lateral sclerosis, Parkinson's disease, and Lewy body dementia,^8^, ^9^, ^27^ and can discriminate between patients with and without AD pathophysiological markers in patients with Lewy body dementia with good diagnostic accuracy.^27^ Thus, CSF studies further support β‐synuclein as a surrogate biomarker of synapse loss people with AD pathophysiology.

Blood‐based biomarkers are of great clinical relevance for the study of AD pathology in adults with DS, given that blood is a more accessible biofluid that allows larger longitudinal clinical studies in this population, and will be essential for screening, disease, and treatment monitoring in future clinical trials. The development of more sensitive techniques for the quantification of β‐synuclein, such as IP‐MS,^11^ has allowed the detection of β‐synuclein in blood. Immunoassays for detecting β‐synuclein have been developed, although their application has primarily been restricted to CSF.^8^, ^28^ A Single Molecule Array‐based assay enabled its detection in blood at the higher concentrations observed in conditions such as Creutzfeldt‐Jakob disease and stroke^26^, ^29^; however, its sensitivity remains limited at the lower concentrations typically observed in AD. To date, there has been little success in validating other synaptic blood biomarkers due to contamination of peripheral tissues. However, β‐synuclein is a promising candidate due to the specificity of expression that is limited to the brain and retina.^30^ This specificity has given rise to multiple studies investigating blood β‐synuclein in different neurodegenerative conditions. In sporadic AD, serum β‐synuclein is increased in mild cognitive impairment and AD dementia compared to controls,^11^, ^23^, ^31^ correlates with temporal brain atrophy and cognitive impairment, and is related to Aβ and tau PET markers.^31^ This study builds on previous reports of elevated serum β‐synuclein in adults with DS at symptomatic compared to asymptomatic AD stages in an independent cohort.^6^ It is also the first study of β‐synuclein that (a) makes a clear differentiation between the symptomatic stages of AD (prodromal and dementia) in DS, showing changes at all AD stages compared to controls, and (b) compares β‐synuclein to biofluid and neuroimaging biomarkers and measures of cognitive performance. Other studies have reported an association of serum β‐synuclein with cognitive impairment in sporadic AD^23^ and in adults with DS and symptomatic AD compared to controls, but not in asymptomatic individuals, and did not stratify dementia versus prodromal stages.^6^

The association in our study of β‐synuclein with cognitive scores and with atrophy of the fusiform gyrus in adults with DS prior to AD symptom onset suggests that this marker could be useful in clinical trials as an additional neurodegeneration marker closely associated with cognitive decline. The strength of association with cognition was mild as could be expected when comparing an objective quantitative assay to a more subjective informant‐based questionnaire. Inclusion of β‐synuclein in AD clinical trials for people with DS and in patients receiving anti‐amyloid therapies could provide further invaluable insight into the clinical utility of this blood‐based biomarker.

A limitation of this study is its cross‐sectional design, which cannot provide information on the trajectory of the biomarkers over time. Longitudinal studies of β‐synuclein in DS are needed to better understand the evolution of these biomarkers through the timeline of the disease. We used LOESS regression to estimate the age at which these biomarkers diverge in DS compared to controls. However, the exact age at which the groups diverge depends on many factors, such as the sensitivity of the assay or the sample size. In this regard, the platforms used for each biofluid marker (IP‐MS for β‐synuclein, immunoassays for pTau217, NfL, and GFAP) have demonstrated high sensitivity in blood.^4^, ^5^, ^11^, ^32^ β‐synuclein was measured in serum, whereas pTau217, NfL, and GFAP were measured in plasma. Recent preliminary data suggest that serum and plasma measurements of p‐tau, NfL, and GFAP are highly correlated.^33^ Therefore, we do not expect the matrix to be a contributing factor to the differences we report for these biomarkers across the AD continuum in DS. In addition, the voxel‐wise analyses were thresholded without correction for multiple comparisons and are therefore exploratory, with the stratified analyses in particular requiring replication in larger cohorts.

In conclusion, this study supports β‐synuclein as a synaptic marker in blood that reflects early synaptic changes in AD pathophysiology in DS, and the early changes observed highlight the added value of β‐synuclein over the existing panel of blood‐based biomarkers in DS before the appearance of clinical AD.

## CONFLICT OF INTEREST STATEMENT

DA participated in advisory boards from Fujirebio‐Europe, Roche Diagnostics, Grifols S.A., Schwabe, and Lilly and received speaker honoraria from Fujirebio‐Europe, Roche Diagnostics, Nutricia, Krka Farmacéutica S.L., Zambon S.A.U., Neuraxpharm, Alter Medica, Lilly, and Esteve Pharmaceuticals S.A. DA, AL, and OB declare holding a patent for markers of synaptopathy in neurodegenerative disease (licensed to ADx NeuroSciences N.V., WO2019175379 Markers of synaptopathy in neurodegenerative diseases). JA has received personal fees for service on the advisory boards, speaker honoraria, or educational activities from Esteve, Lilly, and Roche Diagnostics. JF reported serving on advisory boards and adjudication committees or received speaker honoraria from AC Immune, Adamed, Alzheon, Biogen, Eisai, Esteve, Fujirebio, Ionis, Laboratorios Carnot, Life Molecular Imaging, Lilly, NovoNordisk, Perha, Roche, and Zambón. Daniel Alcolea participated in advisory boards from Fujirebio‐Europe, Roche Diagnostics, Grifols S.A., Schwabe, and Lilly and received speaker honoraria from Fujirebio‐Europe, Roche Diagnostics, Nutricia, Krka Farmacéutica S.L., Zambon S.A.U., Neuraxpharm, Alter Medica, Lilly, and Esteve Pharmaceuticals S.A. AL reported receiving personal fees for service on advisory boards or speaker honoraria from Almirall, Beckman‐Coulter, Biogen, Eisai, Esteve, Fujirebio‐Europe, Grifols, KRKA, Lilly, Novartis, NovoNordisk, Nutricia, Otsuka Pharmaceutical, Roche, and Zambón. AL is co‐author of a patent on antibodies for amyloid precursor, methods and uses thereof European priority (N°EP25382226). MO received consulting fees from Biogen, Axon, Roche, and Grifols and participates on the Biogen ATLAS trial board, all unrelated to the work presented in this paper. PO received consulting fees from LifeArc and Fundamental Pharma, honoraria for lectures from GSK and Fujirebio, and travel support from Biogen. MO and PO are co‐inventors on a patent application for using beta‐synuclein measurement in blood and co‐founders of Neurochem2Dx UG. ACG, AM‐N, SS, BB, LV, IB, SF, LdH, ASH, LMB, LV‐A, IR‐B, MRA, DP, LL, and AB have nothing to disclose. ACG, AM‐N, SS, BB, LV, IB, SF, LdH, ASH, LMB, LV‐A, IR‐B, MRA, and PO contributed to the acquisition and analysis of data. ACG, SS, AM‐N, and OB drafted a significant portion of the manuscript and figures. All authors read and approved the final manuscript. Author disclosures are available in the Supporting Information.

## CONSENT STATEMENT

All participants or their legally authorized representatives gave their written informed consent to participate in the study.

## Supporting information



Supporting information: alz71860‐sup‐0001‐SuppMat.docx

Supporting information: alz71860‐sup‐0002‐ICMJEDisclosureForms.pdf
