# Supplementary material for: Changes in serum β‐synuclein precede blood biomarkers of Alzheimer pathology in Down syndrome
Source: Alzheimers Dement. 2026 Sep 21;22(9):e71860. doi: 10.1002/alz.71860 (PMC13594768; doi:10.1002/alz.71860)
Supplement: Supplementary file 2 [file ALZ-22-e71860-s001.docx]

***Fig S1. Levels of serum β-synuclein and blood-based biomarkers in euploid controls and adults with DS across the AD continuum according to CSF amyloid. (a-f)*** *Box plots show the z-score distribution (mean +/-standard error of the mean) of the biomarkers quantified in blood for non-trisomic cognitively normal subjects (controls) and adults with DS across AD stages; asymptomatic AD negative for CSF amyloid (aDS Aβ-), asymptomatic AD positive for CSF amyloid (aDS Aβ+), prodromal AD (pDS) or AD dementia (dDS). *p<.05, **p<.01, ***p<0.001 (pairwise comparisons from linear models with BH correction).*

***Fig S2. Levels of serum β-synuclein by sex and APOE*** ***ε4 status.*** *Box plots show mean +/- standard error of the mean for serum β-synuclein levels in* ***(A)*** *female and male participants and* ***(B)*** *APOE ε4 carriers (APOE ε4+) and non-carriers (APOE ε4-).*

***Fig S3. Relationship between serum β-synuclein and measures of cognitive performance in DS.*** *Serum β-synuclein levels in adults with DS with mild/moderate intellectual disability are plotted against (A) CAMCOG-DS (B) modified cued immediate recall test (mCRTi), and (C) modified cued delayed recall test (mCRTd), stratifying by clinical status (aDS (orange), pDS (green) and dDS (blue)). Linear regression lines are shown. Shaded areas represent the standard error of the regression lines.*
